# Association of interleukin 1 gene polymorphism with intervertebral disc degeneration risk in the Chinese Han population

**DOI:** 10.1042/BSR20171627

**Published:** 2018-07-06

**Authors:** Yimin Chen, Haitao Ma, Dawei Bi, Binsong Qiu

**Affiliations:** 1 Department of orthopaedics, The First People’s Hospital of Xiaoshan, Hangzhou, 199 Shixin South Road, Xiaoshan district, Hangzhou, Zhejiang, China; 2 Department of Orthopaedics, Zhejiang Provincial People’s Hospital, People’s Hospital of Hangzhou Medical College, 158 Shangtang Road, Hangzhou, Zhejiang, China

**Keywords:** Interleukin-1 alpha, Intervertebral disc degeneration, meta-analysis, single nucleotide polymorphism

## Abstract

Intervertebral disc degeneration (IDD) is a major pathological process implicated in low back pain and is a prerequisite to disk herniation. Interleukin-1 α (IL-1α) was thought to be involved in the pathogenesis of disc degeneration by increasing the production of extracellular matrix degradation enzymes and by inhibiting extracellular matrix synthesis. IL-1α may provide insight about the etiology of IDD. We performed a hospital-based case–control study involving 200 IDD patients and 200 controls in the Chinese Han population. Genotyping was performed using a custom-by-design 48-Plex single nucleotide polymorphism Scan™ Kit. Our study indicated that IL-1α -899C/T polymorphism could increase the risk of IDD under the homozygous, recessive, and allelic models. Subsequently, we validated this significant association by a meta-analysis. Stratification analysis of ethnicity in this meta-analysis also obtained a significant association among Asians and Caucasians. In conclusion, the present study finds that IL-1α -899C/T polymorphism is associated with the risk of IDD. Larger studies with more diverse ethnic populations are needed to confirm these results.

## Introduction

Low back pain (LBP) is a chronic and common medical problem with enormous socioeconomic implications [1]. Eighty-four percent of the population was expected to suffer from LBP at some point in their lifetime [2]. Intervertebral disc degeneration (IDD) was considered as the major cause of clinical LBP, however with an obscure molecular mechanism [3]. It may attribute to genetic and environmental factors (lifting heavy loads, torsional stress, and motor vehicle driving) [4].

Robert et al. [5] found that as IDD proceeds, there are elevated levels of inflammatory cytokines (interleukin 1 (IL-1) and tumor necrosis factor α), enhanced aggrecan and collagen degradation, and changes in disc cell phenotype. IL-1 may contribute to IDD by decreasing the synthesis and increasing the catabolism of proteoglycans [6]. There are three members in the IL-1 gene family: IL-1α, IL-1β, and IL-1 receptor antagonist. The expression of IL-1α was observed in cultured tissues of human aged disc [7]. Moreover, Maeda et al. [8] indicated that the decline in proteoglycan synthesis and increased cell sensitivity to IL-1α with age could contribute to the degeneration of discs.

IL-1α -889C/T polymorphism was associated with level of IL-1α protein [9]. Compared with the CC genotype, the TT genotype of -889C/T polymorphism significantly increased the transcriptional activity of IL-1α gene [10]. Many studies attached importance to the association between IL-1α -889C/T polymorphism and the risk of IDD [11–14]. Karppinen et al. [13] found that this single nucleotide polymorphism (SNP) was associated with the increased risk of IDD in the Finland population. However, this significant association was not observed in the other Caucasian studies [11,12,14]. To date, there is no study exploring the association between IL-1α -889C/T polymorphism and risk of IDD in Chinese Han population. Therefore, we performed the present study involving 200 patients and 200 controls to evaluate whether IL-1α -889C/T polymorphism conferred susceptibility to IDD. In addition, due to the conflicting findings of above studies, it is necessary to perform a meta-analysis combining the present study with those published papers between IL-1α -889C/T polymorphism and IDD risk.

## Materials and methods

### Study subjects

Between March 2014 and January 2017, a total of 200 patients with IDD (103 males and 97 females) and 200 healthy controls (111 males and 89 females) from the First People’s Hospital of Xiaoshan District, Hangzhou were enrolled in the present study. The subjects with a family history of degenerative diseases and biochemical abnormalities did not conform to our inclusion criteria. All cases were diagnosed with IDD according to magnetic resonance imaging (MRI) results [15] and IDD was confirmed by postoperative pathological analyses. Signal intensity changes in vertebra body marrow adjacent to the end plates of degenerative disks are commonly observed on MR images. They appear to take three main forms (Type I, Type II, and Type III) [16]. Type I changes show a decreased signal intensity on T1-weighted images and an increased signal intensity on T2-weighted images; Type II changes present an increased signal intensity on T1-weighted images and an iso- or slightly hyperintense signal on T2-weighted images; Type III changes display a decreased signal intensity on both T1- and T2-weighted images [16]. All postoperative pathological analyses were verified by two experienced pathologists. The healthy controls were individuals receiving the health screening in the same hospital during the same time period. Control groups were matched (1:1) to the IDD cases by sex and age. We collected personal data such as residential regions, age, nationality, sex, height, and weight by an epidemiological questionnaire and in-person interview. All the individuals voluntarily participated in the study and signed an informed consent form before enrollment. The protocol for the present study was approved by the Ethics Committee of the First People’s Hospital of Xiaoshan District, Hangzhou (Hangzhou, Zhejiang, China).

### Determination of genotypes

Following the manufacturer’s protocol, the QIAamp DNA Blood Mini Kit (Qiagen, Hilden, Germany) was used to extract DNA from peripheral blood samples collected from IDD patients and controls. For quality control, repeated analyses were done for 4% of randomly selected samples with high DNA quality. High-throughput MALDI-TOF (Genesky Biotechnologies Inc., Shanghai, China) technology was applied to genotyping. The primers of -889C/T polymorphism used for polymerase chain reaction (PCR) were 5’-GGCTGGCCACAGGAATTAT-3’ (forward); 5’-GCCCAAGGTGTGTCTTCTGT-3’ (reverse). Then we sequenced the PCR products by a MassARRAY 4.0.

### Statistical analysis

Differences between IDD patients and control subjects in term of demographic and clinical characteristics were calculated using the χ^2^ or *t*-test. Odds ratios (ORs) and 95% confidence intervals (CIs) were used to estimate the association between IL-1α gene polymorphisms and risk of IDD by logistic regression analyses. The main homozygous genotypes of IL-1α -889C/T were considered as reference groups for the analysis. A goodness-of-fit χ^2^ test was used to assess whether the genotype distributions of IL-1α -889C/T polymorphism deviated from the Hardy–Weinberg equilibrium (HWE). In order to identify the association of IL-1α -889C/T polymorphism with IDD, we also conducted a meta-analysis. All statistical analyses were performed using the SPSS version 22.0 software package and the Stata 11.0 software (StataCorp, College Station, TX, U.S.A.). All *P*-values presented in the present study were two sided, and *P*<0.05 was considered to indicate a significant difference.

## Results

### Subject characteristics

The final analysis consisted of 200 IDD patients and 200 controls. There were 51.5 and 55.5% men in case and controls (*P*=0.171) respectively. Regarding age and body mass index (BMI), there was no significant difference between the two groups. But it did not hold true in the occupation composition (*P*=0.008). Among the 200 IDD patients, 11 patients were classified as Type I, 38 as Type II, and 151 as Type III according to the signal intensity changes in vertebral body. Most patients are regarded as Pfirrmann Grading 2, 3, and 4. As for the possible relationship between the Pfirrmann grading and the types of MRI, we could not make any further analyses, although they may have an underlying association. The demographics and clinical data of patients with IDD and control subjects are summarized in Table 1.

**Table 1:** Patient demographics and risk factors in intervertebral disc degeneration

Variable	Cases (*n*=200)	Controls (*n*=200)	*P*
Age (years)	42.51 (±4.42)	41.93 (±4.03)	0.171
Male/Female	103/97	111/89	0.423
BMI	22.80 (±2.57)	23.24 (±2.64)	0.088
Occupation			0.008
Taxi driver	97 (48.5%)	84 (42.0%)	
Construction worker	56 (28.0%)	41 (20.5%)	
Office worker	47 (23.5%)	75 (37.5%)	
Signal intensity changes in vertebral body			
^*^Type I	11 (5.5%)		
^†^Type II	38 (19.0%)		
^‡^Type III	151 (75.5%)		
Pfirrmann Grading System			
1	10 (5.0%)		
2	59 (29.5%)		
3	67 (33.5%)		
4	46 (23.0%)		
5	18 (9.0%)		

^*^ a decreased signal intensity on T1-weighted images, and an increased signal on T2-weighted images. ^†^ an increased signal on T1-weighted images, and iso- or slightly hyperintense signal on T2-weighted images. ^‡^ decreased signal intensity on both T1- and T2-weighted images.

### Allele and genotype frequency distributions of IL-1α -889C/T polymorphism

The genotyping frequencies of IL-1α -889C/T polymorphism in cases and controls were summarized in Table 2. Observed genotype frequency distributions of this polymorphism were in agreement with HWE (*P*=0.701). Using logistic regression analysis, we found that the TT genotype of this polymorphism was associated with an elevated risk of IDD (TT vs CC, OR 2.35, 95% CI, 1.16–4.73, *P*=0.017). Moreover, individuals carrying TC + CC genotypes of -887C/T had deceased susceptibility to IDD as compared with the TT genotype (TT vs TC + CC, OR 2.22, 95% CI, 1.13–4.36, *P*=0.021). The significant association was also observed in the allelic model (*P*=0.034).

**Table 2:** Logistic regression analysis of associations between IL-1α -899C/T polymorphism and risk of intervertebral disc degeneration

Genotype	Cases* (*n*=200)		Controls* (*n*=200)		OR (95% CI)	*P*
	n	%	n	%		
TC vs CC	78/87	39.0/43.5	81/102	40.5/51.0	1.13 (0.74,1.73)	0.574
TT vs CC	28/87	14.0/43.5	14/102	7.0/51.0	**2.35 (1.16,4.73)**	0.017
TT+TC vs CC	106/87	53.0/43.5	95/102	47.5/51.0	1.31 (0.88,1.95)	0.186
TT vs TC+CC	28/165	14.0/82.5	14/183	7.0/91.5	**2.22 (1.13,4.36)**	0.021
T vs C	134/252	33.5/63.0	109/285	27.3/71.3	**1.39 (1.03,1.89)**	0.034

Bold values are statistically significant (*P*<0.05).

* The genotyping was successful in 193 cases and 197 controls.

### Meta-analysis of association between IL-1α -899C/T polymorphism and risk of IDD

**Figure 1:**
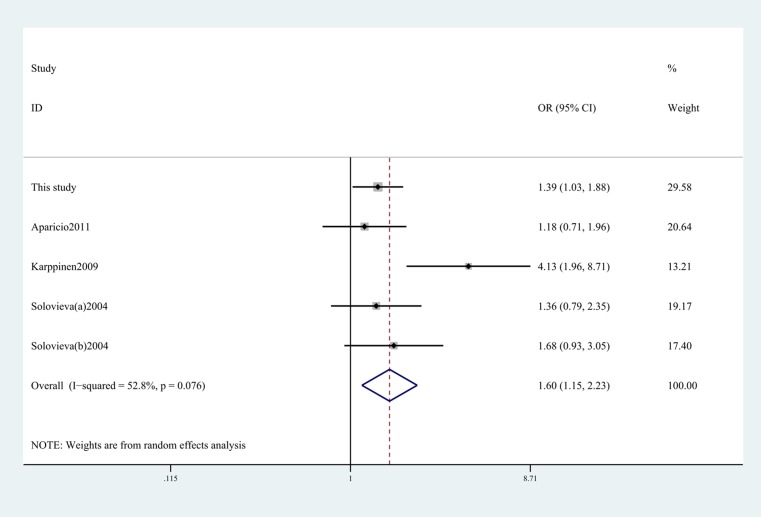
Forest plot shows odds ratio for the associations between IL-1α -889C/T polymorphism and IDD risk (T vs C).

**Figure 2:**
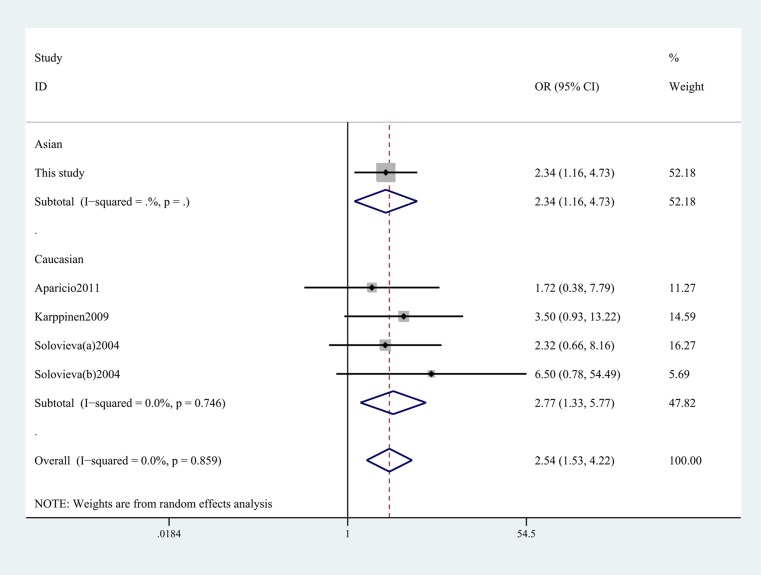
Stratification analyses of ethnicity between IL-1α -889C/T and IDD risk (TT vs CC).

As shown in Table 3, previous studies only studied the association between IL-1α -889C/T polymorphism and risk of IDD among Caucasian populations. The present study attached importance to exploring the relationship in the Asian population. In order to better understand the genetic risk of IL-1α -899C/T polymorphism, we conducted this meta-analysis to illuminate this association. This meta-analysis revealed that IL-1α -899C/T polymorphism was shown to significantly increase IDD risk in the overall populations under allelic (T vs C, OR 1.60, 95% CI, 1.15–2.23, *P*=0.006; Figure 1), dominant, recessive, and homozygous models. In the subgroup analysis of ethnicity, we found that this SNP showed correlation with increased risk among Caucasian populations and Asian populations in the allelic and homozygous models (TT vs CC, OR 2.77, 95% CI, 1.33–5.77, *P*=0.003; Figure 2) (Table 4).

**Table 3:** Characteristics of included studies

Author and year	Country	Ethnicity	Case			Control			NOS
			CC	TC	TT	CC	TC	TT	
Solovieva (a) 2004	Finland	Caucasian	11	21	6	34	51	8	7
Solovieva (b) 2004	Finland	Caucasian	30	54	13	15	18	1	7
Karppinen 2009	Finland	Caucasian	12	26	7	30	2	5	6
Aparicio 2011	Spain	Caucasian	22	25	3	63	61	5	7
Present study	China	Asian	87	78	28	102	81	14	6

Abbreviations: NOS, Newcastle–Ottawa Scale

**Table 4:** Meta-analysis of association between IL-1α -899C/T polymorphism and the risk of IDD

Comparison	Category	Category	Studies	OR (95% CI)	*P*-value	*P* for heterogeneity
T vs C	Total		5	**1.60 (1.15, 2.23)**	0.006	0.076
	Ethnicity	Asian	1	**1.39 (1.03, 1.88)**	0.034	N/A
		Caucasian	4	**1.73 (1.07, 2.80)**	0.025	0.046
TT + TC vs CC	Total		5	**1.94 (1.05, 3.61)**	0.035	0.004
	Ethnicity	Asian	1	1.31 (0.88, 1.95)	0.186	N/A
		Caucasian	4	2.29 (0.95, 5.51)	0.065	0.003
TT vs TC + CC	Total		5	**2.07 (1.29, 3.32)**	0.003	0.792
	Ethnicity	Asian	1	**2.21 (1.13, 4.36)**	0.021	N/A
		Caucasian	4	1.92 (0.98, 3.76)	0.056	0.677
TT vs CC	Total		5	**2.62 (1.59, 4.32)**	<0.001	0.859
	Ethnicity	Asian	1	**2.34 (1.16, 4.73)**	0.017	N/A
		Caucasian	4	**2.77 (1.33, 5.77)**	0.003	0.746
TC vs CC	Total		5	1.82 (0.91, 3.65)	0.093	0.002
	Ethnicity	Asian	1	1.13 (0.74, 1.72)	0.574	N/A
		Caucasian	4	2.35 (0.84, 6.56)	0.103	0.001

Bold values are statistically significant (*P*<0.05)

## Discussion

The present study revealed that IL-1α -889C/T polymorphism was associated with an increased risk of IDD in the Chinese Han population. It also held true in the overall populations by a meta-analysis. Subgroup analysis by ethnicity revealed increased risk for Caucasians and Asians.

IDD is a major pathological process implicated in LBP and is a prerequisite to disk herniation [17]. Few studies have explored the role of IL-1α in the etiology of IDD in the last 3 years. In 2017, Cai et al. [18] found that levels of IL-1α were higher in degenerated disc tissue-conditioned medium (dCM) compared with the control medium. The catabolic effect of dCM on healthy nucleus pulposus cells is mediated by MAPK and NF-κ B pathways and can be reduced by TGF-β1 [18]. In addition, Li et al. [19] demonstrated that the level of IL-1α in degenerated lumbar disc tissues are higher than normal, and the increasing levels are positively correlated with the disease condition. IL-1α was thought to be involved in the pathogenesis of disc degeneration by increasing the production of extracellular matrix degradation enzymes and by inhibiting extracellular matrix synthesis [12, 20]. Moreover, some SNPs of IL-1α have showed correlation with a risk of IDD [21,22]. IL-1α -889C/T polymorphism was associated with level of IL-1α protein [9] and transcriptional activity of IL-1α gene [10].

Several studies have studied the association between IL-1α -889C/T polymorphism and IDD risk previously [11–14]. Karppinen et al. [13] found that IL-1α -889C/T polymorphism showed correlation with IDD risk in the Finland population. The remaining studies failed to replicate this positive association among other Caucasian populations [11,12,14]. Different sample sizes, low statistical power, and/or potential genetic heterogeneity may account for these disparities of above studies. It is of note that these identified studies investigated diverse types of IDD, such as symptomatic lumbar disc herniation, LBP, and intervertebral disc degeneration. We hypothesized that above clinical heterogeneity may explain the different findings of these studies. Meta-analysis integrates single studies and increases statistical power and resolution by pooling the results of individual studies. In order to overcome the limitations of individual studies, Wang et al. [23] conducted a meta-analysis with 330 cases and 419 controls to evaluate the effect of IL-1α -889C/T polymorphism on IDD risk in 2016. Their results revealed that IL-1α -889C/T polymorphism increased the risk of IDD among Caucasian populations in the most genetic comparisons. Notably, Wang et al. [23] included an original case–control study by Serrano et al. [24], which did not meet their inclusion criteria actually. Serrano et al. [24] explored the association between IL-1α gene rs1800587 polymorphism and IDD risk, but not IL-1α -889C/T polymorphism. Therefore, we should interpret the results of Wang et al. with caution. The present study suggested that T allele or TT genotype of IL-1α -889C/T polymorphism could increase the risk of IDD in Chinese Han population. We re-evaluated the role of IL-1α -889C/T polymorphism in the risk of IDD by a meta-analysis combining the present study with 423 cases and 490 controls and found it was associated with increased risk for IDD. The finding of this meta-analysis is almost in line with that of the previous meta-analysis [23]. Some potential limitations of the present study need to be addressed. One, the statistical calculations were conducted without other risk factors, such as smoking and drinking. Two, the sample size of the present study was not large enough, which might make our work underpowered. Three, IDD is a multifactorial disease, and thus the function of a single SNP may be restricted. Four, included studies of this meta-analysis were mainly involved in Caucasians, and only one Asian study was included; thus, other ethnicity should be further investigated to validate these findings. Fifth, the present study only explore IL-1α -889C/T polymorphism; however, other IL-1α gene SNPs are warranted.

In summary, IL-1α -889C/T polymorphism may be associated with an increased risk of IDD in Chinese Han population. Meta-analysis further indicated significant association in the overall populations. More studies with larger sample sizes are warranted to determine whether IL-1α -889C/T polymorphism is associated with IDD risk.

## Abbreviations

BMI: body mass index
CI: confidence interval
dCM: disc tissue-conditioned medium
HWE: Hardy–Weinberg equilibrium
IDD: intervertebral disc degeneration
IL-1: interleukin-1
IL-1α: interleukin-1 alpha
LBP: low back pain
MRI: magnetic reson
ORs: odds ratios
PCR: polymerase chain reaction
SNP: single nucleotide polymorphism
